# Erysipelas

**Published:** 1853-10

**Authors:** 


*Erysipelas.*—Dr. Balfour has treated twenty cases of this disease, with tincture of sesquichloride of iron, and believes, that we have now "a certain and unfailing remedy, whether the erysipelas be infantile or adult, idiopathic or traumatic." The dose is ten to twenty minims every two hours, preceded by a cathartic.—*St. Louis Med. and Surg. Jour.*

